# Drug-induced senescence bystander proliferation in prostate cancer cells *in vitro* and *in vivo*

**DOI:** 10.1038/sj.bjc.6604288

**Published:** 2008-03-18

**Authors:** J A Ewald, J A Desotelle, N Almassi, D F Jarrard

**Affiliations:** 1 Department of Urology, University of Wisconsin School of Medicine and Public Health, Madison, WI 53792, USA; 2 University of Wisconsin Paul P Carbone Comprehensive Cancer Center, Madison, WI 53792, USA; 3 University of Wisconsin Environmental and Molecular Toxicology Program, 600 Highland Avenue, Madison, WI 53792, USA

**Keywords:** senescence, bystander effect, prostate cancer, proliferation

## Abstract

Senescence is a distinct cellular response induced by DNA-damaging agents and other sublethal stressors and may provide novel benefits in cancer therapy. However, in an ageing model, senescent fibroblasts were found to stimulate the proliferation of cocultured cells. To address whether senescence induction in cancer cells using chemotherapy induces similar effects, we used GFP-labelled prostate cancer cell lines and monitored their proliferation in the presence of proliferating or doxorubicin-induced senescent cancer cells *in vitro* and *in vivo*. Here, we show that the presence of senescent cancer cells increased the proliferation of cocultured cells *in vitro* through paracrine signalling factors, but this proliferative effect was significantly less than that seen with senescent fibroblasts. *In vivo*, senescent cancer cells failed to increase the establishment, growth or proliferation of LNCaP and DU145 xenografts in nude mice. Senescent cells persisted as long as 5 weeks in tumours. Our results demonstrate that although drug-induced senescent cancer cells stimulate the proliferation of bystander cells *in vitro*, this does not significantly alter the growth of tumours *in vivo*. Coupled with clinical observations, these data suggest that the proliferative bystander effects of senescent cancer cells are negligible and support the further development of senescence induction as therapy.

Senescence is a physiological programme of terminal growth arrest occurring in both normal and immortalised cells in response to telomeric alterations, and also to sublethal stress and inappropriate oncogenic signalling. Senescent cells develop a characteristic phenotype, including an enlarged, flattened morphology, prominent nucleus, senescence-associated heterochromatin foci (SAHF), and senescence-associated *β*-galactosidase (SA*β*-gal) activity (Narita *et al*, 2003; Campisi, 2005; Lee *et al*, 2006). Cancer treatments, including radiation and chemotherapy, induce senescent characteristics in cells. Doxorubicin and cisplatin are more efficient in generating senescence in cell culture than ionising radiation, etoposide or the microtubule-targeting drugs docetaxel and vincristine (Chang *et al*, 1999). Heterogeneous SA*β*-gal staining has been observed in sections of frozen human breast tumours after treatment with cyclophosphamide, doxorubicin and 5-fluorouracil (te Poele *et al*, 2002), and in lung tumours, after carboplatin and taxol (Roberson *et al*, 2005). Senescence develops at lower drug concentrations than apoptosis, potentially limiting treatment-related side effects (Schwarze *et al*, 2005).

Senescence may provide a number of unique therapeutic benefits. When senescence is induced by expressing p53 in a murine liver cancer model, an upregulation of inflammatory cytokines triggers an innate immune response that targets the tumour cells (Xue *et al*, 2007). Other studies have suggested senescence may function as an alternative mechanism of tumour inhibition. In mice bearing E*μ*-myc lymphomas, treated with cyclophosphamide, when apoptosis was blocked by Bcl-2 overexpression, senescence developed and these animals had improved survival over the apoptotic tumours (Schmitt *et al*, 2002). The recognition that a senescence programme may be reinduced in immortalised and tumorigenic cells by exposure to selected drugs presents a putative target for blocking cancer cell growth.

However, senescence induction may potentially promote tumour growth. Senescent cells express a variety of growth factors and secreted proteins that may stimulate as well as inhibit cell proliferation (Chang *et al*, 2002; Schwarze *et al*, 2002, 2005; Untergasser *et al*, 2002; Bavik *et al*, 2006). In contrast to apoptosis, a programme of cellular destruction, senescent cells persist and remain viable. SA*β*-gal activity in cells has been putatively identified in ageing tissues, including skin and benign prostatic hyperplasia specimens (Dimri *et al*, 1995; Choi *et al*, 2000). Consistent with the hypothesis that ageing induces a procarcinogenic environment, fibroblasts passaged to replicative senescence induce the proliferation of local bystander cells both *in vitro* and in xenografts (Krtolica *et al*, 2001; Bavik *et al*, 2006). To determine whether senescent cancer cells generate a bystander effect or not, we chemically induced senescence in prostate cancer cells using doxorubicin and examined their effect on a bystander cancer cells *in vitro* and *in vivo*.

## MATERIALS AND METHODS

### Cell lines and cell culture

DU145 and LNCaP prostate cancer cell lines, and human primary fibroblasts, were cultured and senescence induced by treatment with 25 nM doxorubicin as described previously (Schwarze *et al*, 2005). Polyclonal green fluorescence protein (GFP)^(+)^ cell lines were generated by infecting DU145 and LNCaP cells with pLS-GFP virus and repeated sorting of GFP^(+)^ cells. Resulting cell lines stably express GFP in ∼98 and ∼80% of DU145- and LNCaP-derived cell lines, respectively. GFP^(+)^ cells in both lines were approximately 100 × brighter than non-labelled cells, as measured by flow cytometry (data not shown).

### Cell-counting experiments

For coculture experiments, 50 000 DU145 or 200 000 LNCaP GFP^(+)^ tagged cells and equivalent proliferating or senescent untagged cells or 50 000 senescent primary prostate fibroblasts were plated together in triplicate in 35-mm wells containing growth medium. The following day, cells were washed twice in phosphate-buffered saline (PBS), given minimal medium (50% F12/50% DMEM+penicillin/streptomycin) and returned to 37°C, 5% CO_2_. Cells were collected after 2 or 4 additional days in culture. Cell viability in counted samples was determined by annexinV binding (Invitrogen, Carlsbad, CA, USA) and by propidium iodide exclusion. Data were acquired from samples by flow cytometry and analysed using WinMDI v2.8 software (Joseph Trotter, Scripps Research Institute) to calculate the total number of viable GFP^(+)^ cells in each sample.

Counting experiments were repeated using threefold the number of proliferating or senescent cells (from 50 000 to 150 000 cells), or a decreased fraction of senescent cocultured cells (75 and 25% senescent *vs* proliferating), incubated in minimal medium for 4 days and analysed as above.

### BrdU incorporation

In cell-counting experiments (above), 20 mM BrdU was added to cell-culture medium, 30 min prior to trypsinisation, and GFP^(+)^ cells were recovered by fluorescence-activated cell sorting. Isolated cells were fixed in 100% ethanol and stored at −20°C. Subsequently, cells were rehydrated and stained for BrdU as described previously (Krtolica *et al*, 2001; Schwarze *et al*, 2003). BrdU incorporation of cells cocultured in transwells did not require cell sorting.

### Xenograft cocultures

All animal protocols and studies were conducted in accordance with the guidelines of the Association for Assessment and Accreditation of Laboratory Animal Care International, and approval was obtained from the University of Wisconsin Institutional Animal Care and Use Committee. Male athymic nude mice were obtained from Harlan (Madison, WI, USA). Xenograft tumours were established as described previously (Passaniti *et al*, 1992a, 1992b). DU145-GFP^(+)^ and unlabelled proliferating or senescent DU145 cells (0.5 × 10^6^, each) were injected into the mouse subinguinal fat pad and allowed to develop into xenograft tumours over 5 weeks time. Tumour dimensions were measured at 3, 4 and 5 weeks after injection using a caliper. BrdU was injected into these mice interperitoneally at a concentration of 70 mg kg^−1^ body weight (Christov *et al*, 1993), harvested 2 h later and dissociated into a single-cell suspension from which GFP^(+)^ cells were isolated by fluorescence-activated cell sorting. These were fixed in ice-cold ethanol and stored at −20°C. BrdU incorporation was measured in recovered cells, as mentioned above.

LNCaP xenografts were established by injecting 1 × 10^6^ LNCaP cells alone, with 50% Matrigel (BD Biosciences, San Jose, CA, USA) or with an equal number of senescent LNCaP cells as described (Passaniti *et al*, 1992a, 1992b), and cells were measured as mentioned above. Additionally, xenografts were established using 0.5 × 10^6^ DU145 cells with or without addition of equal number of senescent GFP^(+)^-DU145 cells. Tumours were measured as mentioned above, harvested at 3 and 5 weeks and samples were frozen in OCT for sectioning.

### Immunofluorescence staining and microscopy

Ten micrometre sections of xenografts were fixed in PBS+4% paraformaldehyde/0.2% Triton X-100/10 mM NaF/1 mM Na3VO_4_ and washed in PBS+0.2% Triton X-100/10 mM NaF/1 mM Na3VO_4_ (wash buffer) before incubation in blocking buffer (wash buffer + 10% fetal bovine serum +1% bovine serum albumin) for 1 h at room temperature. Sections were washed in blocking buffer and incubated with 1 *μ*g ml^−1^ anti-IGF2 as a cellular counterstain (1 : 200 dilution; Santa Cruz Biotechnology, Inc., Santa Cruz, CA, USA; no. sc-5622) overnight at 4°C. Sections were again washed, incubated for 1 h with 200 ng ml^−1^ (1 : 1000 dilution) anti-rabbit-Alexa 594+10 ng ml^−1^ Hoechst 33342 (Invitrogen), washed and mounted using ProLong Gold (Invitrogen). Images were captured using an Olympus microscope with mercury lamp, appropriate filters and spot digital camera and imaging software (Diagnostic Instruments Inc., Sterling Heights, MI, USA). Images were merged and visualised using NIH ImageJ (http://rsb.info.nih.gov/ij/).

### Statistical methods

Data were analysed, standard deviation and standard error were calculated, and Student's *t*-tests were performed using Microsoft Excel. Error bars in all figures represent one standard deviation in the data.

## RESULTS

**Figure 1:**
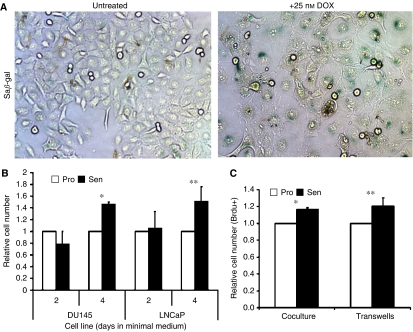
Proliferative bystander effect of drug-induced senescent prostate cancer cells *in vitro*. (**A**) Bright-field images of DU145 cells cultured on cover slips ±25 nM doxorubicin (DOX) for 3 days, fixed and stained for SA *β*-gal activity (400 ×). (**B**) Number of proliferating DU145-GFP^(+)^or LNCaP-GFP^(+)^ cells after coculture with proliferating or senescent non-tagged cancer cells measured by flow cytometry. Replicate results were averaged from four independent experiments. These results represent the average fold increase of cell numbers in senescent cocultures relative to proliferative cell data. Error bars represent standard error (^*^P<0.0001; ^**^P=0.022). (**C**) BrdU+ incorporation in cells after direct coculture (left) and in transwells (right) after 30 min incubation with 20 uM BrdU. The results of three independent experiments were averaged and the numbers of cells from senescent cocultures were normalised to that of proliferating cocultures. Error bars represent standard error (^*^P=0.003, ^**^P<0.0001).

We generated stable GFP-expressing lines of the hormone-refractory DU145 (p53-inactive) and the androgen-dependent LNCaP (expressing functional p53) prostate cancer cell lines. To monitor the bystander effect of chemically induced senescent cancer cells, GFP^(+)^ cells were cocultured with proliferating or senescent unlabelled cancer cells, collected and analysed by flow cytometry. Both DU145 and LNCaP cells treated with low-dose (25 nM) doxorubicin for 3 days develop a senescent phenotype, increased SA*β*-gal staining (Figure 1A), and express previously described senescence marker genes (Schwarze *et al*, 2005).

Initially, DU145-GFP^(+)^ or LNCaP-GFP^(+)^ cells were plated with equal numbers of proliferating or doxorubicin-induced senescent untagged cells and cultured in a minimal serum-free medium for 2 and 4 days. GFP^(+)^ cells cocultured with senescent cells were similar in number to those cocultured with proliferating cells at 2 days (Figure 1B). However, after 4 days, a significant increase in DU145 (1.46 fold; *P*<0.0001) and LNCaP (1.51 fold; *P*=0.022) cells was observed when cocultured with senescent cells. Apoptosis of GFP^(+)^ cells, measured by annexin-V binding and propidium iodide exclusion at each time point, was not significantly affected by the presence of senescent cells (<1% in each sample), suggesting that these observed differences were not due to effects on cell survival. Proliferation, measured by BrdU incorporation, was also increased at day 4 (16–21%; *P*=0.003) in GFP^(+)^ DU145 cells, exposed to senescent cells (Figure 1C). When DU145 and LNCaP cells were cocultured in 0.4 m transwell inserts, preventing contact between the two populations but allowing exposure to common media, BrdU incorporation was similarly increased (20–24%; *P*<0.0001 and *P*<0.05, respectively). Given the similar magnitude of this proliferative response to the mixing experiments performed on single plates suggested the majority of the growth stimulation observed was induced by secreted soluble factors.

Increasing the numbers of cocultured proliferating and senescent cells threefold in both DU145 and LNCaP cells (150 000 cells) sustained this proliferative response (1.4-fold; in both DU145 and LNCaP cells *P*=0.03 and *P*=0.003, respectively; data not shown) demonstrating that this effect was not an artifact of media depletion. Decreasing the fraction of cocultured senescent cells to 38 and 12% of the total cell population (decrease of 25 and 75% in the unlabelled senescent cells) did not induce proliferation (data not shown). These results demonstrate that a proliferative bystander effect can be stimulated *in vitro* by chemically induced senescent prostate cancer cells through paracrine signalling.

**Figure 2:**
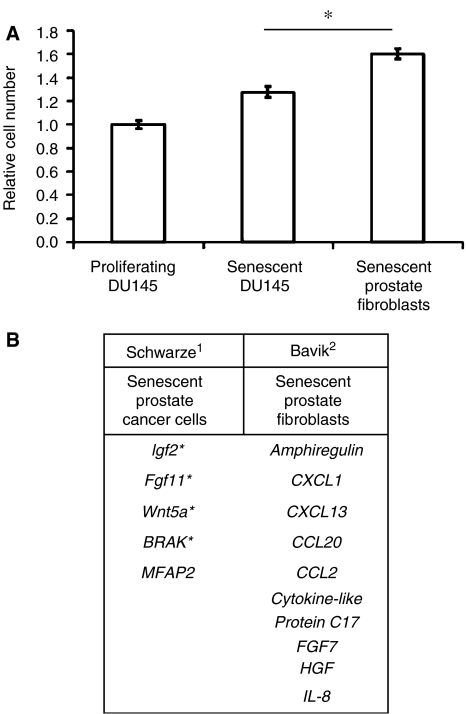
Bystander proliferation induced to a greater extent by replicatively senescent prostate fibroblasts than senescent prostate cancer cells. (**A**) Number of proliferating DU145-GFP^(+)^ cells cocultured with proliferating or senescent DU145 cells or three independent primary prostate fibroblast cell lines after passage to replicative senescence. Data from all three senescent fibroblast lines were averaged. Results are expressed relative to proliferating coculture data. Error bars represent standard error (^*^P<0.01). Results are representative of two experiments. (**B**) Expression of secreted growth factor genes reported in chemically induced senescent prostate cancer cells and senescent fibroblast (^1^Schwarze *et al*, 2005 *Neoplasia*; ^2^Bavik *et al*, 2006. *Canc. Res.* ^*^Increased gene expression confirmed in senescent cancer cells by quantitative RT-PCR in the present study).

Previously published data have demonstrated a significant proliferative response of bystander cells to senescent fibroblast lines (Krtolica *et al*, 2001; Bavik *et al*, 2006). Therefore, we compared the proliferative bystander response of senescent DU145 cells to three replicatively senescent prostate fibroblast lines generated through prolonged passage in cell culture (Figure 2A). Senescent fibroblasts demonstrated SA-*β* gal staining and senescent morphology. After 4 days in coculture, the increase in the number of prostate cancer cells exposed to senescent fibroblasts was twice that seen with senescent cancer cells (60 *vs* 30%, respectively; *P*<0.01). We then confirmed the induction (>2 fold) of a number of growth-promoting paracrine factors in our chemically induced senescent DU145 and LNCaP cells (Figure 2B) using qPCR. No increase in expression of these genes *(IGF2, BRAK, FGF11* and *Wnt5a*) was seen in the senescent fibroblast lines. Comparing growth-promoting gene expression data from a number of studies involving fibroblasts, epithelial cells and cancer cells (Schwarze *et al*, 2005; Bavik *et al*, 2006) reveals little overlap when fibroblasts are compared to other cell lines (Figure 2B). In sum, our data show that senescent fibroblasts induce the proliferation of bystander cells *in vitro* significantly more than senescent prostate cancer cells.

Next, we investigated whether senescent cancer cells promote the growth of non-senescent cancer cells in nude mouse tumour xenograft models or not. LNCaP prostate cancer xenografts require additional growth factors, provided by Matrigel™, to establish viable tumours and proliferate (Passaniti *et al*, 1992a, 1992b). To determine if senescence has a similarly permissive effect on xenograft tumour establishment, mice were injected with 1 × 10^6^ LNCaP cells either alone, with 50% Matrigel or with 1 × 10^6^ senescent LNCaP cells (*n*=5 in each group). Six weeks after injection, LNCaP cells coinjected with Matrigel developed into viable tumours in all five animals. In contrast, tumours did not develop under the other conditions (0/10 mice). This demonstrates that chemically induced senescent LNCaP cells do not promote tumour establishment and/or growth of this cell line.

**Figure 3:**
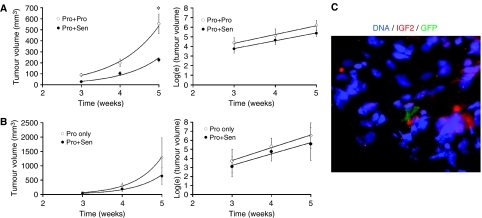
Xenograft tumour growth is not promoted by senescent DU145 cells. (**A**) Average size (left) and natural log of tumour size (right) of prostate xenograft tumours established using DU145-GFP^(+)^ cells (0.5 × 10^6^) mixed with an equal number proliferating (Pro+Pro) or senescent (Pro+Sen) cells and measured for 5 weeks. Error bars represent standard error (^*^P<0.001). Fit equations: (left) (Pro+Pro): *y*=5.491e^0.913*x*^ (*R*^2^=0.996); (Pro+Sen): *y*=1.362e^1.039*x*^ (*R*^2^=0.981); (right) (Pro+Pro): *y*=0.917*x*+1.565 (*R*^2^=0.989); (Pro+Sen): *y*=0.813*x*+1.340 (*R*^2^=0.997). (**B**) Average size (left) and natural log of tumour size (right) of prostate xenograft tumours established using DU145 (0.5 × 10^6^) cells alone (Pro only) or with an equal number of additional senescent GFP^(+)^-DU145 cells (Pro+Sen). Error bars represent standard error. Fit equations: (left)(Pro only): *y*=0.739e^1.491*x*^ (*R*^2^=0.999); (Pro+Sen): *y*=0.576e^1.417*x*^ (*R*^2^=0.994); (right)(Pro only): *y*=1.41*x*−0.458 (*R*^2^=0.994); (Pro+Sen): *y*=1.254*x*−0.527 (*R*^2^=0.966). (**C**) Xenograft tumour section containing a senescent GFP^(+)^-DU145 cell. Hoechst 33342 (blue) was used to stain nuclei and anti-IGF2 (red) was used to stain cytoplasm (400 ×). This image is representative of sections from 10 xenograft tumours containing 1–4 GFP^(+)^ cells per section (mean: 1 hpf).

Next, we examined the effect of senescent cells on tumour growth in DU145 xenografts using two different approaches. First, we coinjected 0.5 × 10^6^ DU145-GFP^(+)^ proliferating cells with an equal number of unlabelled proliferating or senescent DU145 cells (1 × 10^6^ total) to model the effects of treatment-induced senescence in 50% of tumour cells. Tumours were palpable in both groups after 2 weeks and tumour dimensions were measured 3, 4 and 5 weeks after injection. The average volume of tumours established with or without senescent cells was calculated for each time point. Reflecting the greater number of proliferating cells initially injected, xenografts containing only proliferating cells grew significantly larger than those containing senescent cells after 5 weeks (*P*<0.001) (Figure 3A, left). However, the average exponential rate of tumour growth was not significantly affected by the presence of senescent cells, illustrated by calculating the natural log (ln) of the average tumour volume over time (Figure 3A, right). Control animals, in which only senescent cells were injected, did not develop palpable tumours through the course of these experiments. Mice were injected with 70 mg kg^−1^ body weight BrdU 2 h prior to tumour harvest to measure proliferation in sorted GFP^(+)^ tumour cells (Christov *et al*, 1993). Cells from DU145 tumours established with or without senescent cells and collected after 5 weeks contain similar fractions of proliferating cells as measured by BrdU uptake and DNA profiling (data not shown). As a second approach, we repeated this experiment by beginning with equivalent numbers (0.5 × 10^6^) of proliferating DU145 cells and determining the effect of adding additional (0.5 × 10^6^) senescent cells. Again, the presence of senescent cells did not increase average tumour size or the rate of tumour growth (Figure 3B left, right).

Using senescent GFP^(+)^-DU145 cells in this second approach allowed us to determine whether senescent cells persisted through the growth of these tumours or not. GFP^(+)^ senescent cells were detected in xenograft tumours harvested 3 and 5 weeks after injection. However, at these time points, senescent cells were found infrequently (1–4 cells per section; mean 1 hpf; Figure 3C). SA *β*-gal analysis of tumour sections demonstrated infrequently stained cells, confirming these findings (data not shown). These results demonstrate that non-proliferating senescent cells become diluted during xenograft growth, yet persist even 5 weeks after injection. Therefore, the presence of chemically senescent cancer cells does not increase the rate of xenograft tumour establishment or growth *in vivo*.

## DISCUSSION

Significant interest has been generated regarding the role of senescence as a tumour suppressor and the clinical ramifications of its reactivation in cancer (Schmitt *et al*, 2002; Petti *et al*, 2006; Xue *et al*, 2007). Potential exists for development of therapeutic compounds that specifically induce senescence in cancer cells (Roninson, 2003). However, concerns have been raised regarding the promoting effect of senescent cancer cells on the tumour microenvironment, similar to that seen with senescent fibroblasts (Kahlem *et al*, 2004). Our results demonstrate that a limited proliferative response occurs *in vitro* with chemically induced senescent cells when compared to senescent fibroblasts (Figure 2A). However, this bystander effect does not affect xenograft tumour establishment or the growth of non-senescent bystander tumour cells *in vivo* (Figure 3).

Using multiple cell types and combinations, senescent cells did not impact *in vivo* tumour growth or proliferation. When xenografts were established using proliferating cells with and without senescent cells, tumours were consistently smaller in the presence of senescent cells (Figure 3B). We acknowledge that a transient increase in proliferation may be induced prior to the development of a palpable tumour, but clearly, the long-term impact on tumour size was not significant. Technically similar mixing experiments in immune-deficient mice have been performed using senescent fibroblasts and a stimulatory effect was easily demonstrated using multiple immortalised and tumorigenic cell lines (Krtolica *et al*, 2001; Parrinello *et al*, 2005; Bavik *et al*, 2006). *In vivo*, these studies utilised equivalent numbers of proliferating and senescent cells similar to our methods. Our data clearly show the lack of a stimulatory response when senescent cancer cells are mixed with proliferating cancer cells in tumours. Furthermore, with current chemotherapy regimens, senescent cells appear at a much lower frequency (<20%) than those tested in our experiments (te Poele *et al*, 2002; Roberson *et al*, 2005).

As part of our study, we contrasted, *in vitro*, the bystander effect of senescent fibroblasts to that seen with chemically induced senescent cancer cells. Using our quantitative and reproducible model, the *in vitro* proliferative effect of senescent cancer cells was noted to be 40–50% of that seen with senescent fibroblasts. Our quantitative PCR analysis confirmed the results of studies that show significant variation between growth-promoting genes expressed by senescent epithelial cells, fibroblasts and cancer cells (Chang *et al*, 2002; Schwarze *et al*, 2002, 2005; Untergasser *et al*, 2002; Zhang *et al*, 2003; Bavik *et al*, 2006). The finding that gene expression perturbations during senescence differ greatly between fibroblasts and epithelial cells, but show physical clustering on DNA, has been thought to reflect the altered chromatin structure seen during senescence (Zhang *et al*, 2003). These changes are likely to be even more marked in cancer cells containing deletions, duplications and distorted nuclear structure.

*In vivo*, the expression of secreted extracellular matrix, growth factors and surface receptor proteins differs markedly from cells cultured *in vitro* (Gieseg *et al*, 2004). This disparity in the tumour microenvironment may contribute to the lack of induction of proliferation in response to senescent cells *in vivo*. As an example, IGF2 protein expression is clearly elevated in senescent cancer cells *in vitro*, but the expression of IGF2 protein does not quantitatively differ *in vivo*, when senescent and proliferating cells are compared (data not shown). A unique aspect of our study is the demonstration of a persistence of senescent cells in tumours as long as 5 weeks after injection. They represent a small population at this time point, less than 1%, due to expansion of the proliferating population, which doubles in roughly 48 h (Passaniti *et al*, 1992a, 1992b). Senescent cells have been noted in the skin of elderly individuals (Dimri *et al*, 1995) and in melanocytic naevi (Michaloglou *et al*, 2005). Our data in a xenograft model would support the persistence of these cells in various organs.

Placing senescence induction in the context of cancer treatment, our results suggest that the specific induction of senescence in prostate tumour cells would not promote tumour growth. Accumulating data suggest that senescent cells may occur *in vivo* after the treatment of tumours with chemotherapy, in approximately 40% of breast tumours after treatment using a CAF regimen (te Poele *et al*, 2002). Other observations support that senescence *in vivo* is a beneficial phenotype by inducing a cellular immune response (Petti *et al*, 2006; Xue *et al*, 2007) and demonstrating a survival advantage when compared to solely apoptotic responses (Schmitt *et al*, 2002). Recently, senescent cells were identified in human melanocytic nevi, a benign, stable skin lesion, supporting its function as a long-term tumour-suppressive mechanism (Michaloglou *et al*, 2005). In this case, there are no apparent signs of enhanced bystander proliferation or increased local carcinogenesis. Staining for senescent cells has also been identified in benign prostatic hyperplasia tissues, a common benign entity not associated with cancer (Choi *et al*, 2000). In conclusion, our data demonstrate that the presence of chemically senescent prostate cancer cells does not significantly enhance the growth of tumour xenografts, providing further rationale for the development of anticancer strategies that efficiently induce senescence in advanced cancers.
